# Semi‐intensive shrimp farms as experimental arenas for the study of predation risk from falcons to shorebirds

**DOI:** 10.1002/ece3.8059

**Published:** 2021-09-07

**Authors:** Enzo Basso, Mark C. Drever, Juanita Fonseca, Juan G. Navedo

**Affiliations:** ^1^ Bird Ecology Lab Instituto de Ciencias Marinas y Limnológicas Universidad Austral de Chile Valdivia Chile; ^2^ Programa de Doctorado en Ecología y Evolución Facultad de Ciencias Universidad Austral de Chile Valdivia Chile; ^3^ Environment and Climate Change Canada Pacific Wildlife Research Centre Delta British Columbia Canada; ^4^ Facultad de Ciencias del Mar Universidad Autónoma de Sinaloa Mazatlán México; ^5^ Western Hemisphere Shorebird Reserve Network Executive Office, Manomet Mazatlán México

**Keywords:** anthropogenic habitats, aquaculture, foraging, peregrine falcon, tidal amplitude, vigilance behavior, western sandpiper

## Abstract

Varying environmental conditions and energetic demands can affect habitat use by predators and their prey. Anthropogenic habitats provide an opportunity to document both predation events and foraging activity by prey and therefore enable an empirical evaluation of how prey cope with trade‐offs between starvation and predation risk in environments of variable foraging opportunities and predation danger. Here, we use seven years of observational data of peregrine falcons *Falco peregrinus* and shorebirds at a semi‐intensive shrimp farm to determine how starvation and predation risk vary for shorebirds under a predictable variation in foraging opportunities. Attack rate (mean 0.1 attacks/hr, equating 1 attack every ten hours) was positively associated with the total foraging area available for shorebirds at the shrimp farm throughout the harvesting period, with tidal amplitude at the adjacent mudflat having a strong nonlinear (quadratic) effect. Hunt success (mean 14%) was higher during low tides and declined as the target flocks became larger. Finally, individual shorebird vigilance behaviors were more frequent when birds foraged in smaller flocks at ponds with poorer conditions. Our results provide empirical evidence of a risk threshold modulated by tidal conditions at the adjacent wetlands, where shorebirds trade‐off risk and rewards to decide to avoid or forage at the shrimp farm (a potentially dangerous habitat) depending on their need to meet daily energy requirements. We propose that semi‐intensive shrimp farms serve as ideal “arenas” for studying predator–prey dynamics of shorebirds and falcons, because harvest operations and regular tidal cycles create a mosaic of foraging patches with predictable food supply. In addition, the relatively low hunt success suggests that indirect effects associated with enhanced starvation risk are important in shorebird life‐history decisions.

## Graphical abstract

Shrimp farms can serve as ideal arenas for the study of trade‐offs between starvation and predation risk in shorebird populations at coastal areas during the nonbreeding season.

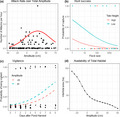

## 1 INTRODUCTION

Predation affects prey species through direct and indirect effects (Cresswell, 2008). Direct effects reduce survival rates of prey populations, while indirect effects include behavioral changes by prey to reduce predation risk, which may carry other costs (Lima, 1998). For instance, prey may avoid suitable habitats and forage in food‐poor but less hazardous areas or allocate more time to antipredator strategies such as gathering in large flocks or increasing vigilance behavior to the detriment of feeding time (Beauchamp & Ruxton, 2008; Cresswell & Quinn, 2011; Lima & Dill, 1990). Additionally, varying environmental conditions (e.g., temperature) and high energetic demands during some periods of the annual cycle (e.g., migration) can affect habitat use by prey and, consequently, the spatial scale of direct and indirect effects (Cresswell, 2008; Yasué et al., 2003). Thus, during periods of high‐energy requirements, prey must trade off between risk of starvation and predation and feed in profitable but dangerous habitats (Houston & McNamara, 1999; Lima & Dill, 1990). This starvation–predation trade‐off in vertebrates is well known, with most studies measuring energy management in prey exposed to different experimental set‐ups (see recent work in Broggi et al., 2019; Monarca et al., 2020). However, mainly because of the limitations imposed by the logistics of observing predation events in the wild, particularly in systems involving birds both as predators and prey, the study of predator–prey dynamics would benefit from the identification of opportunities to document both predation events and how prey cope with this trade‐off in environments of predictable food supplies.

Migratory shorebirds (mainly Families Scolopacidae and Charadriidae) face different levels of danger along the annual cycle (Ydenberg et al., 2007), with raptor predation being an important cause of mortality both in staging/stopover and nonbreeding areas (Dekker & Drever, 2016; Whitfield, 2003). Shorebirds respond to high predation risk by raptors in a variety of ways, including body mass reduction (Piersma et al., 2003) and modifying timing of primary molt and wing length (Lank et al., 2017; Ydenberg et al., 2007). Additionally, under the Flight Early to Avoid Rush (FEAR) hypothesis (Blumstein, 2010), shorebirds display antipredator behaviors that rely on flock size (such as confusion/dilution effects or enhanced vigilance) to reduce per‐capita predation risk (Cresswell & Quinn, 2011). However, vulnerability may increase under energetic stress, when individuals must allocate less time to antipredator behaviors to increase overall intake rate (Lima, 1998). Thereby, shorebirds under starvation risk may be forced to forage in profitable but dangerous habitats by feeding in smaller group sizes, consequently reducing their ability to detect attacking raptors (Quinn & Cresswell, 2004), thus resulting in higher predation risk.

Periods of consecutive days under high energetic stress are periodically experienced by shorebird populations. To survive during the nonbreeding season, shorebirds rely on crucial habitats such as coastal wetlands (Davidson & Evans, 1986), where tides constrain availability of foraging areas on predictable daily (high and low tides) and monthly (neap and spring tides) cycles (Calle et al., 2016). When feeding opportunities are restricted, for example, during high tides and during neap tide periods (Fonseca et al., 2017), shorebirds may use artificial wetlands such as salinas and aquaculture ponds not limited by tidal cycles (Masero, 2003) to maintain their high daily energy requirements (Smart & Gill, 2003). Indeed, several studies have highlighted the potential role of artificial wetlands as important trophic subsidies for migratory shorebird populations (Fonseca & Navedo, 2020; Masero, 2003; Weber & Haig, 1996). However, shorebirds should trade off risk of starvation and predation if they face higher predation risk at these artificial habitats that could reduce survival (Yasué et al., 2003). Hitherto predation risk at these anthropogenic habitats has primarily been indirectly assessed by studies of prey behavior (Barbosa, 1997; Rosa et al., 2006), rather than measuring predation rate, and remains poorly understood (Dwyer et al., 2013).

In northwestern Mexico, semi‐intensive shrimp farms (following Edwards, 1993) enmeshed within surrounding coastal wetlands are widely used by shorebirds to forage throughout the shrimp‐harvesting period between October and December (Navedo & Fernández, 2019; Navedo et al., 2015). These anthropogenic habitats represent ideal arenas to study trade‐offs between starvation and predation risk from a complete ecological perspective for a number of reasons. First, high densities of Nearctic shorebirds forage at recently harvested ponds, even at those shrimp farms adjacent to or embedded with large coastal wetlands (Navedo & Fernández, 2019). Second, predators as peregrine falcons (*Falco peregrinus*) are often observed hunting on shorebirds at shrimp farms (Basso et al., 2018). Third, tidal cycles offer predictable varying foraging opportunities and restrictions at nearby intertidal areas depending of daily tidal amplitude (Fonseca et al., 2017). Finally, use of shrimp farms by shorebirds varies in a highly predictable manner following shrimp harvest (Navedo et al., 2017), where high densities of invertebrates (particularly Nereidae polychaetes; De León‐González et al., 2018) become available as the water levels are lowered and pond bottom is exposed (Fonseca & Navedo, 2020). For example, prey biomass for shorebirds decreased from 1.4 to 0.8 g AFDW·m^−2^, equivalent to a 43% reduction, over three days after shrimp harvest (Fonseca & Navedo, 2020). Given shorebird use mirrors this temporal pattern of water levels and food availability (Navedo et al., 2017), semi‐intensive shrimp farms provide a sort of natural experiment to study prey–predator interactions throughout the harvesting period, enabling an assessment of how shorebirds manage predation risk under semi‐controlled conditions of food availability.

**FIGURE 1:**
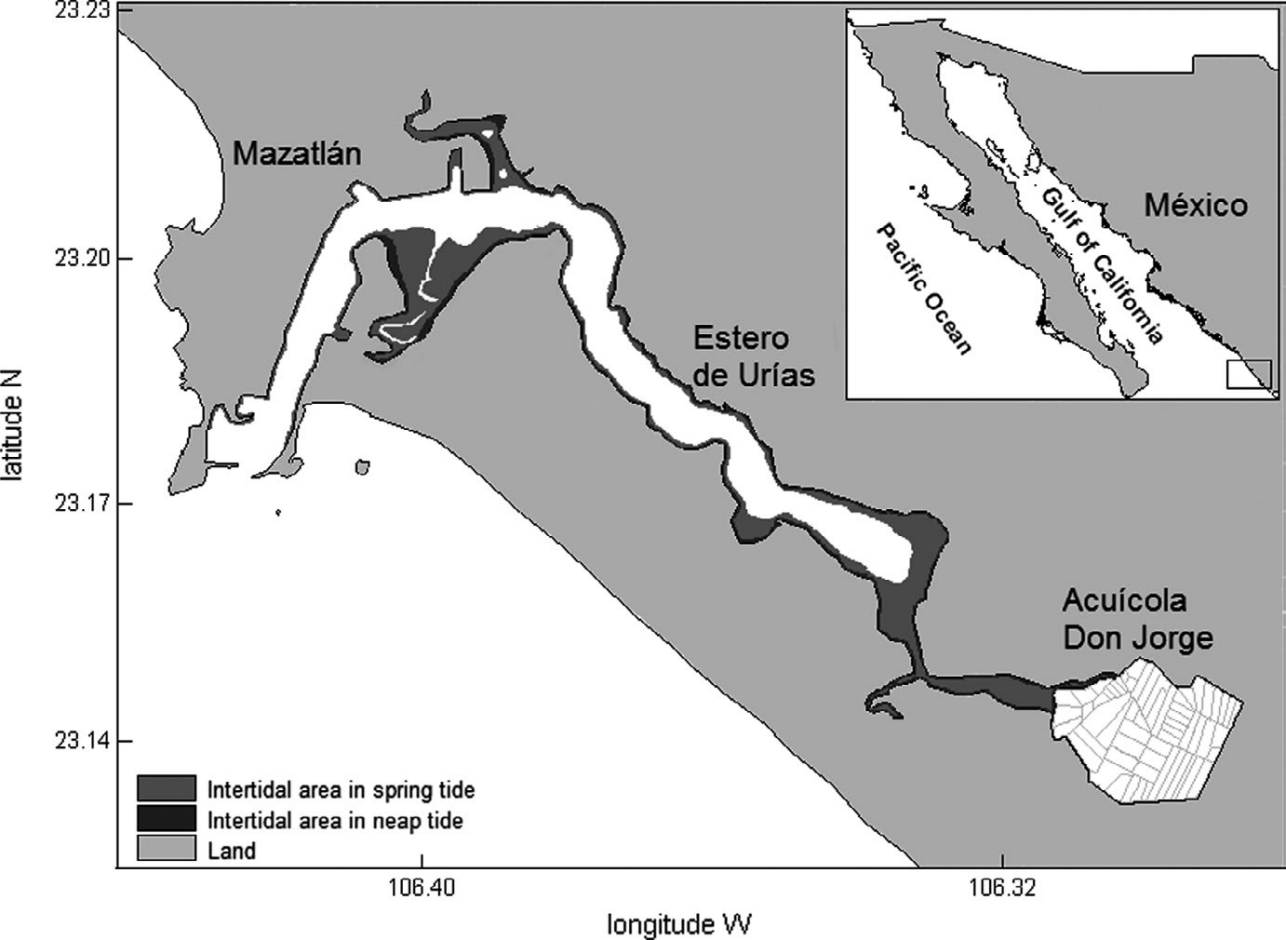
Location of Acuícola Don Jorge shrimp farm and its proximity to Estero de Urías, a coastal lagoon, in northwestern Mexico. Within the coastal lagoon, light gray shows overall intertidal areas exposed during spring tides and dark gray areas those exposed during neap tide periods (based on Fonseca et al., 2017)

Here, we evaluated predator–prey interactions between peregrine falcons and Nearctic shorebirds in a semi‐intensive shrimp farm associated with a coastal lagoon in the northwest of Mexico. This study focuses on the predation risk to shorebirds relative to conditions at the shrimp farm within its surrounding wetland complex, which includes both the farm and the adjacent intertidal areas (Figure 1), as part of an ongoing pioneer research program examining shorebird use of an operational shrimp farm (Basso et al., 2018; Fonseca & Navedo, 2020; Navedo et al., 2015, 2017). We hypothesized that shorebirds move between alternative foraging habitats, balancing actual foraging opportunities and predation danger. If a threshold exists, then shorebirds will use dangerous areas when tidal conditions restrict availability of tidal habitats and create a scenario of low foraging opportunities (i.e., higher risk of starvation) to meet daily energy requirements. Using behavioral observations collected during seven field seasons, we calculated the frequency of attacks by peregrine falcons and measured hunt success and vigilance behavior by shorebirds under different conditions of food availability in recently harvested ponds. If predation danger is high, then we predicted that (a) attack rate would increase during periods when foraging opportunities at nearby intertidal habitats are restricted due to tidal conditions, and higher numbers of shorebirds use the shrimp farm to forage, thus attracting predators. In addition, (b) hunt success would be higher during low‐tide periods, when shorebird numbers at the shrimp farm are lower than during high tide (Basso et al., 2018), and thus chances of detection of an approaching predator as well as dilution and confusion effects would be reduced due to smaller flock sizes (Blumstein, 2010). Finally, because flock sizes decreased in parallel with food availability after 3–4 days once a pond is harvested (Fonseca & Navedo, 2020; Navedo et al., 2017), then (c) individual shorebirds would try to minimize predation risk by increasing vigilance while foraging in such ponds. Our study thus improves our understanding of ecological processes driving predator–prey interactions in animal populations.

## 2 METHODS

### 2.1 Study area

Estero de Urías is a wetland complex located on the northwestern Mexican coast along the Gulf of California (23°11′N, 106°22′W; Figure 1). It has an area of 11 km^2^ with a maximum depth of 21 m (in the mouth) and average depth of 3.4 m (Montaño‐Ley et al., 2008). This wetland presents diverse habitats such as intertidal mudflats, emergent brackish marshes, and surrounding mangrove fringes (*Rizophora mangle*), and is classified as a coastal lagoon of low energy (Lankford, 1977). The tide is predominantly mixed semidiurnal, with a tidal range of 1.20 m (during this study) and form number (K1 + O1)/(M2 + S2) of 0.576 (Fonseca et al., 2017). The wetland has limited freshwater discharges, with salinity range of 25.8–38.4 g/kg (Montaño‐Ley et al., 2008). Available intertidal foraging areas for shorebirds at Estero de Urías are mainly restricted to two areas (Navedo et al., 2015), covering 103.7 ha during spring tides, being reduced to 18.5 ha during neap tides (see Fonseca et al., 2017). To evaluate how daily foraging opportunities for shorebirds varied as a function of tidal amplitude, we calculated the availability of intertidal habitat using the bathymetric matrix from Fonseca et al. (2017). The intertidal area was estimated for each amplitude of 10 cm, according to the maximum and minimum values of the tidal tables for Mazatlán city provided by the CICESE (http://predmar.cicese.mx/calendarios/).

Don Jorge shrimp farm covers 300 ha and is divided in c. 50 ponds averaging 4.7 ha (Figure 1; Navedo et al., 2015). The production system is semi‐intensive, operating in a typical annual growing cycle that lasts between 120 and 140 days (Páez‐Osuna et al., 2003), with two harvests per year. The second harvesting period usually lasts 40 days in this shrimp farm, beginning in October–November. During harvesting, ponds are lowered in water depth and become available for shorebirds to forage. Pond harvesting is sequential (i.e., one to three ponds each day) and consists of pond draining and gathering the shrimps. The quality of ponds as foraging areas dramatically decreases day by day as ponds dry out. In general, ponds provide foraging grounds for shorebirds for 1–5 days after harvest (Navedo et al., 2017). After that period, the substrate dries out and hardens, preventing access for shorebirds to invertebrate prey. Some patches within ponds may remain available after these initial days, but shorebirds substantially deplete polychaetes larger than 40 mm in just three days after pond harvesting (Fonseca & Navedo, 2020), thus significantly reducing available biomass and subsequent pond profitability as foraging grounds. Foraging opportunities at each pond was thus characterized using “pond day” as a proxy, which was based on the number of days since shrimp harvest occur at the focal pond (day of harvest = pond day 0).

### 2.2 Field methods

Within the shrimp farm, we conducted shorebird counts at ponds in different conditions relative to its day of harvest (Navedo et al., 2017). A total of 171 shorebird surveys were conducted during seven field seasons from 2011 to 2019 (14 to 32 survey days per year; median 27 survey days). Predator–prey interactions during daylight hours were assessed during a total of 746 hr of observations, with a mean (±*SE*) of 5.4 ± 0.8 hr per survey day. We focused on recently harvested ponds (up to three days after pond harvesting), since shorebirds densities are very low from day 4 onwards (Navedo et al., 2017). All surveys were conducted by JGN and EB, using 20–60× spotting scopes and 10 × 42 binoculars.

We estimated the frequency of falcon occurrence within the shrimp farm as an overall measure of predation danger. During each survey day, we noted the presence of peregrine falcons perching on poles or on the dikes separating ponds. We noted 0 when no detection was made during a survey and 1 when we observed falcons at least once, either perched or attacking. Following Cresswell and Whitfield (1994), we considered an attack as a direct and fast flight toward one or a group of birds. Multiples chases of the same target during one attack were considered the same event. An attack was considered finished when we observed the peregrine falcon moving away from shorebirds. If another attack was observed some minutes later (minimum time‐lapse between two consecutive attacks considered independent, 5 min), it was considered a new attack. For each attack event, we recorded the hour and duration of the attack, the species composition of the target flock, overall flock size and whether the attack was solitary or cooperative between more than one falcon, and the eventual fate of each attack. An attack was considered successful when a prey was captured by the peregrine falcon. When possible, we noted age‐class of peregrine falcon (i.e., juvenile or adult) and subspecies (based on White et al., 2020).

During two of these seasons (2012 and 2019) when an HDD video camera was available, we took video recordings of foraging behavior of western sandpipers (*Calidris mauri*; the main prey; see results) throughout the harvesting cycle. Following Pomeroy (2006), we estimated vigilance rate as the probability that the individual adopted vigilance behavior at least once along a foraging sequence (mean 44 s, *SD* = 16.2 s, total *n* = 108 sequences). Vigilance behavior typically involved a cessation of foraging, lifting the head, and movements interpreted as a scan for an approaching predator. Foraging sequences when any other disturbance was observed (mainly associated with shrimp harvesting in nearby ponds) were not included. Since western sandpiper densities steeply decline from day two after pond harvesting (Fonseca & Navedo, 2020), we used “pond day” as a proxy to evaluate the effect of flock size as well as food availability on vigilance rate.

To obtain a measure of annual shorebird abundance (i.e., prey) at the entire wetland complex, we carried out a series of repeated low‐tide counts at intertidal areas of the lagoon and at the shrimp farm simultaneously. Censuses were conducted during spring tides to minimize the potential influence of tidal amplitude in overall shorebird abundance at Estero de Urías (Fonseca et al.,  2017). We conducted two or three comprehensive surveys during each year during the harvest period (see full details in Navedo et al., 2015).

### 2.3 Statistical analyses

#### 2.3.1 Attack rate

We evaluated the frequency of attacks at the shrimp farm using a generalized mixed‐effects model, fit with package *lme4* in R (Bates et al., 2015). We reasoned that daily frequency of attacks might vary due to conditions both within the shrimp farm and at the nearby wetland complex. For conditions within the shrimp farm, we evaluated the frequency of attacks as a function of the sum total pond area that was in the state of active or recent harvest (pond days 0, 1, 2) within the entire farm. Ponds in this condition are used by a wide diversity of shorebirds (Fonseca & Navedo, 2020; Navedo et al., 2017). For the nearby wetland complex, we used the tidal amplitude for each survey day, which would determine the timing and amount of mudflats being exposed and thus available for foraging shorebirds (Basso et al., 2018; Fonseca et al., 2017). Tidal amplitude indicates monthly lunar period (spring/neap) and was considered as a variable ranging from −40 to 60 cm in relation to Mean Lower Low Water (0 cm). Counterintuitively, negative values (spring tides) indicate higher availability of nearby intertidal areas, and positive values (neap tides) indicate a lower availability of intertidal foraging areas for shorebirds. We used the total number of attacks observed during each survey as the response variable in the model, and explanatory variables included the number of hours spent during each survey (log‐scaled) as an offset to account for variable survey effort, along with total pond area available to forage and tidal amplitude on each survey date as fixed effects. Given the complex relationship between food availability and tidal amplitude (Basso et al., 2018; Fonseca et al., 2017), we modeled amplitude as a quadratic function to allow a consideration of non‐linear patterns of attack frequency in relation to monthly tidal cycles. All models included year as a random effect, with a log link function, and were fit assuming a Poisson error distribution.

We conducted a post hoc test to evaluate whether the mean annual frequency of falcon attacks was related to overall abundance of western sandpipers (its main prey, see results) throughout the harvest season within the whole wetland complex. Using censuses from 2011 to 2019, we tested whether the random effects of year from the mixed effect model were correlated with the annual average count of western sandpipers.

#### 2.3.2 Hunt success

We evaluated whether the probability of success varied as a function of the following: predator attributes, including the number of falcons (solitary or cooperative), and age‐class; shorebird foraging conditions, including tidal amplitude, tide height (high or low), and pond quality for shorebirds (“optimal”: in harvest days 0, 1, or 2, or “nonoptimal”: 3 or more days after harvest); and prey attributes, including total flock size of all shorebirds, and whether the flock included (yes or no) western sandpipers (the most common shorebird attacked; see Basso et al., 2018). For these analyses, we focused on the 71 occasions during which an attack was observed and classified as 0 if the attack did not result in a prey being caught or 1 if the hunt was successful. Using the *glm* procedure in R, we fit a binomial generalized linear model (GLM) that included all the factors in a global model and then used the *step* procedure to choose a model in a stepwise search that considered both forward and backward search modes from a null model that included only an intercept. Once we identified the variables that warranted inclusion based on a likelihood ratio test (LRT), we refitted a final model with only those retained variables. Given the relatively low number of attacks, we used an alpha‐level of 0.01 to have an inclusive consideration of factors that affect hunt success and considered them in an exploratory way.

#### 2.3.3 Vigilance

We evaluated vigilance rate as the probability that western sandpipers exhibited vigilant behavior at least once during the focal scans. We considered a vigilant behavior when a focal individual stops foraging and raises its head, usually turning it to scan, for some seconds before flushing or resume foraging. Using a binomial GLM, we fit a model that expressed vigilance as 1 or 0, depending on whether vigilant behavior was observed, and which included total duration of scan (in sec) as an offset, with pond day and tidal amplitude as explanatory variables.

## 3 RESULTS

### 3.1 Predator abundance

Peregrine falcons were observed at the shrimp farm during every harvest season throughout the study, with median of two individuals, ranging from a single individual (2012) up to four (2015) different ones (Table 1). We were able to identify either *F.p.anatum* or *F.p.tundrius,* with all juvenile falcons belonging to *F.p.tundrius*. Presence of peregrines was regular at the shrimp farm, with an average (±*SD*) frequency of occurrence of 0.41 ± 0.17 (range 0.11–0.61) during 4–6 hr surveys across the seven year period.

**TABLE 1:** Number of different individual peregrine falcons present each season and daily frequency of occurrence during 4 to 6 hr surveys at a shrimp farm in NW Mexico throughout the harvesting period

Occurrence	2011	2012	2015	2016	2017	2018	2019
Individuals	3	1	4	2	2	1	2
Frequency	0.36	0.11	0.61	0.57	0.29	0.50	0.44

### 3.2 Attack rate

**FIGURE 2:**
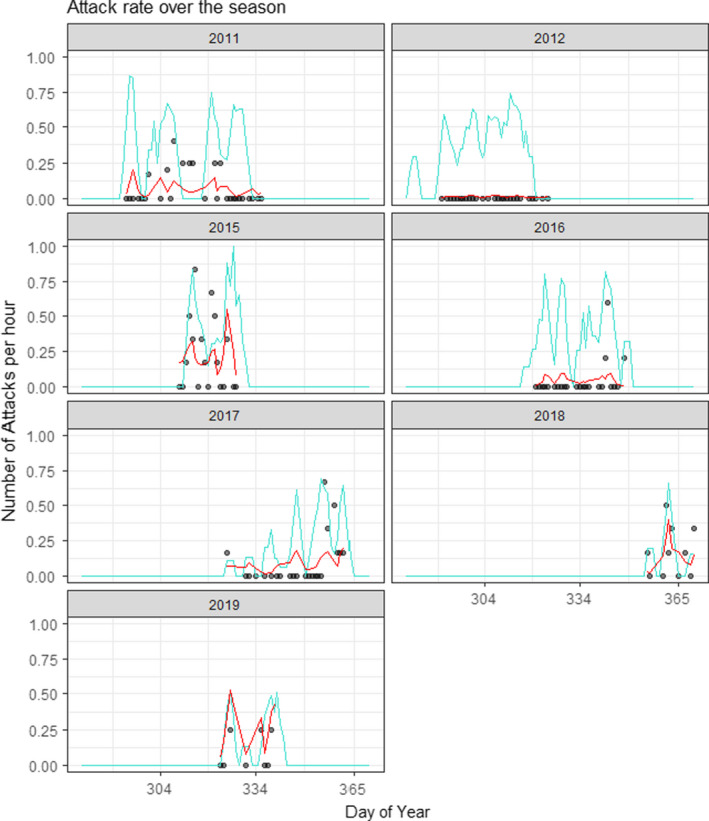
Frequency of attacks by peregrine falcons on shorebirds at Acuícola Don Jorge, a shrimp farm in northwestern Mexico, 2011 to 2019. Black points indicate number of attacks observed per hour, and red lines indicate the predicted values of a model of attack frequency as a function of tidal amplitude and total area of shrimp ponds in a state of recent harvest. Turquoise lines indicate total area of recently harvested shrimp ponds; pond area is scaled to 1 by dividing daily available foraging area by the maximum total daily available foraging area observed in the study period (54.7 ha). One data point of value = 1.75 attacks/hr (tidal amplitude = 28, date = 2019‐12‐06) is not shown

**FIGURE 3:**
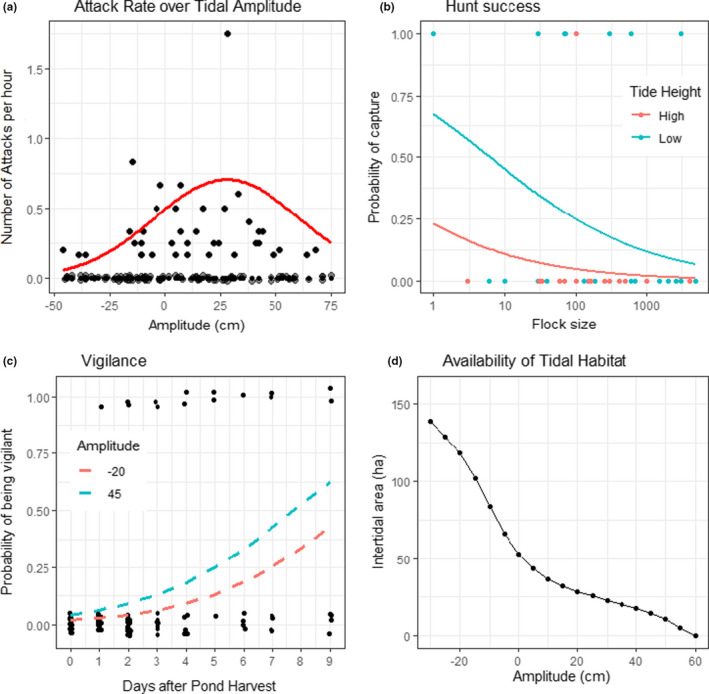
Predator/prey dynamics at Acuícola Don Jorge, a shrimp farm in northwestern Mexico, 2011 to 2019, vary with conditions on the adjacent estuary. (a) Frequency of attacks by peregrine falcons on shorebirds as function of tidal amplitude in the adjacent estuary. Note that negative values indicate higher tidal amplitudes (i.e., spring) and positive values lower ones (i.e., neap). Black points indicate number of attacks observed per hour, with a jitter of 0.01 applied to the 0 values. Red line depicts predicted values of model of attack frequency as a function of tidal amplitude and total area of recently harvested shrimp ponds, with pond area set to its median value. (b) Probability of success of hunts by peregrine falcons on shorebirds. Lines indicate predicted values of a binomial model of hunt success as a function of tide height and size of target flock. Note *x*‐axis is on log_10_ scale. (c) Vigilance behavior exhibited by western sandpipers as a function of harvest state of shrimp ponds and tidal amplitude. Lines indicate predicted values of a binomial model of vigilance as a function of tidal amplitude and number of days after shrimp harvest. Availability of food for shorebirds on shrimp farm ponds declines sharply after harvest. (d) Availability of intertidal habitats for shorebirds to forage at the adjacent estuary as a function of tidal amplitude

Over the seven seasons, we observed 71 attacks by raptors on shorebirds, all of them involving peregrine falcons. Of these, 55 (77.5%) were on flocks of western sandpipers, and the remaining 16 attacks (22.5%) were on 14 other shorebird species, among which black‐necked stilt *Himantopus mexicanus*, willet *Tringa semipalmata*, and whimbrel *Numenius phaeopus* were the most common (Table 2). The frequency of attacks varied widely over time and among years. Attack rate varied from 0 to 1.8 attacks per hour, with a mean of 0.1 attacks per hour (Figure 2). The highest attack rate occurred in 2015 and the lowest in 2012 when no attacks were observed during the entire season. Attack rate was positively associated with the total daily pond area that was in the state of being recently harvested (i.e., optimal condition for shorebirds to forage) at the shrimp farm (*β_p_*_ond_._area_ = 0.023, *SE* = 0.008, *t* = 2.75, *p* = .006; Figure 2). Tidal amplitude had a strong nonlinear effect on the frequency of attacks (*β*_amplitude_ = 0.021, *SE* = 0.007, *t* = 3.15, *p* = .002, *β*_amplitude_^2^ = −0.0006, *SE* = 0.0002, *t* = −3.47, *p* = .005; Figure 3a), indicating a peak in attack rate in‐between spring and neap tide periods. We conducted a follow‐up analysis that separated attacks on shorebird flocks that had western sandpipers from attacks on other shorebird species (e.g., larger‐bodied birds) and found that tidal amplitude and total pond area being harvested had a similar positive effect on both types of shorebird flocks, and therefore, all attacks were considered together.

**TABLE 2:** Frequency of bird species attacked by peregrine falcons on a shrimp farm in northwestern Mexico

Scientific name	Common name	Freq.	Percent (%) of total	Freq. successful
*Calidris mauri*	Western sandpiper	55	77.5	7
*Himantopus mexicanus*	Black‐necked stilt	11	15.5	1
*Tringa semipalmata*	Willet	9	12.7	1
*Numenius phaeopus*	Whimbrel	6	8.5	0
*Tringa melanoleuca*	Greater yellowlegs	6	8.5	0
*Limosa fedoa*	Marbled godwit	4	5.6	1
*Tringa flavipes*	Lesser yellowlegs	3	4.2	0
*Charadrius semipalmatus*	Semipalmated plover	2	2.8	0
*Limnodromus* spp.	Dowitcher spp.	2	2.8	0
*Numenius americanus*	Long‐billed curlew	2	2.8	0
*Egretta thula*	Snowy egret	1	1.4	1
*Gelochelidon nilotica*	Gull‐billed tern	1	1.4	0
*Hydroprogne caspia*	Caspian tern	1	1.4	0
*Leucophaeus atricilla*	Laughing gull	1	1.4	1
*Recurvirostra americana*	American avocet	1	1.4	0

Note

A total of 71 attacks were observed during 171 bird surveys during seven years from 2011 to 2019. The total frequency does not equal 71 because some attacks involved flocks composed of multiple species.

Average annual counts of western sandpipers at the wetland complex varied from 0 birds in 2012 to 10,977 birds in 2015. These annual counts were strongly correlated with the year random effects from the model of attack frequency (*ρ* = 0.90, *n* = 7, *p* = .006), indicating that the annual variation in attack rate was related to the overall abundance of sandpipers during each shrimp harvest season.

### 3.3 Hunt success

Of the 71 attacks observed at the shrimp farm (41 at low‐tide and 30 at high tide), six were cooperative attacks between two falcons. Twelve attacks resulted in prey capture (17%, or 14% for those only considering shorebirds as prey, Table 2), while 57 attacks were unsuccessful (80%), and the fate of two were unknown (3%). The 12 successful hunts involved western sandpipers (*n* = 7), with other prey species (black‐necked stilt, willet, marbled godwit *Limosa fedoa*, snowy egret *Egretta thula*, and laughing gull *Leucophaeus atricilla*), each having one successful occurrence (Table 2). Of the factors considered, hunt success was most strongly related to tide height at time of attack, and prey flock size. Model selection identified these two variables warranted inclusion relative to a null model (LRT Chi^2^ values >3.6, *df* = 1, *p* < .06) and were included in the final model. The parameter for tide period (*β* = 1.92, *SE* = 1.13, *t* = 1.70, *p* = .09) indicated that higher hunt success tended to occur during low tides, while the parameter for flock size (*β* = −0.40, *SE* = 0.19, *t* = −2.06, *p* = .04) indicated the probability of success declined as the target flock became larger (Figure 3b). Neither type of attack nor falcon age‐class had significant explanatory power into hunt success model.

### 3.4 Vigilance

A total of 108 focal observations of western sandpipers were conducted at ponds in the early days after being harvested (pond day 0 to 9 days). On average, 14% of birds exhibited vigilant behavior at least once during focal scans. Noticeably, no sandpipers exhibited vigilant behavior during focal observations when foraging on a pond immediately after shrimp harvest (pond day = 0). The probability of vigilance was positively correlated with pond day (*β* = 0.40, *SE* = 0.12, *t* = 3.4, *p* < .001; Figure 3c), such that by pond day 9, ~50% of birds exhibited vigilant behavior during scans. Probability of vigilance was also positively, but not significantly, associated with tidal amplitude (*β* = 0.01, *SE* = 0.01, *t* = 1.1, *p* = .28).

## 4 DISCUSSION

Our results provide empirical evidence of a trade‐off between starvation and predation risk for shorebirds using an alternative supratidal area to forage during the nonbreeding season. Attacks by peregrine falcons were mainly focused on western sandpipers and varied over time depending on harvest operations at the shrimp farm, being more frequent at recently harvested ponds that also support higher shorebird densities (Navedo et al., 2017). Attack rate on shorebirds at the shrimp farm also varied with conditions at the nearby wetland, showing a gradual increase as the tidal amplitude became smaller and overall foraging opportunities at intertidal areas are severely reduced (Fonseca et al., 2017; Figure 3d). Attack rate then steeply dropped during the neap tide periods, underscoring a non‐linear relationship with tidal conditions. In addition, mean attack rate (0.1 attacks per hour) was within the range observed by peregrine falcons on shorebirds in other coastal areas (0.04–0.75 attacks per hour; Cresswell, 1994, 1996; Cresswell & Whitfield, 2008; Dekker & Drever, 2016) and was highly correlated with the overall abundance of western sandpipers, its main prey, in the wetland. Similarly, seven out of 10 successful attacks on shorebirds involved western sandpipers, and the observed 14% hunt success was similar to previous studies with peregrine falcons and other raptors on shorebirds, which typically varies from 7% to 30% (van den Hout et al., 2008). These results support the consideration that our study system of peregrine falcons and western sandpipers inhabiting a semi‐intensive shrimp farm during the harvesting period can serve as an ideal arena to study predator–prey interactions at coastal areas.

Tides affect the daily availability of mudflats for shorebirds to forage and meet daily energy requirements at coastal habitats (Granadeiro et al., 2006). In our system, the availability of intertidal foraging areas is greatly reduced during neap tides, such that the abundance of western sandpipers in the adjacent wetlands decreased on average from ~1,100 individuals during spring tides to ~200 individuals during neap tides, with no difference in foraging activity (Fonseca et al., 2017). During spring tides, sandpipers seem to meet their daily energetic requirements at intertidal areas and remain there, and therefore, few attacks occur at the shrimp farm. As the tidal amplitude gradually decreases, available foraging opportunities at the adjacent intertidal areas also decrease (Fonseca et al., 2017; Figure 3d); thus, more individuals need to compensate by foraging at the shrimp farm to meet daily requirements. Attack rate by peregrines increased during these periods. Western sandpipers seem thus to tolerate the risk of predation at the shrimp farm in order to supplement their requirements not obtained at intertidal areas when their overall risk of starvation remains low. After a threshold of tidal amplitude that severely restricts available foraging intertidal habitats continuously for three or four days during neap tide peak periods, sandpipers leave the intertidal area entirely (Fonseca et al., 2017). Noticeably, during neap tides, a much lower abundance of sandpipers is also observed at the shrimp farm (Basso et al., 2018), and attack rate by peregrines decreases (Figure 3a). All these results suggest that a risk threshold exist, modulated by a predictable tidal amplitude that determines overall food availability (and therefore starvation risk). Shorebirds trade‐off risk and rewards to avoid or forage at the shrimp farm depending on whether an individual can achieve its daily energy requirement by supplementary foraging in a potentially dangerous habitat.

Once at the shrimp farm, shorebirds can reduce per‐capita predation risk by aggregating into large flocks that can reduce hunt success by peregrine falcons via increased vigilance, dilution, and confusion effects (Cresswell & Quinn, 2010). Foraging in large groups is an important antipredator strategy employed by shorebirds to deal with the trade‐off between starvation and predation risk (Cresswell & Quinn, 2010, 2011; Stinson, 1980). The shared vigilance that occurs in larger group sizes could increase the probability that western sandpipers detect an attack of peregrine falcon early and consequently reduces the individual vigilance time, enabling more time to forage (Beauchamp, 2001; Elgar, 1989). Multiple targets in movement (i.e., confusion effect) could reduce the efficacy of open opportunistic attacks of peregrine falcons, thereby decreasing the hunt success (Cresswell et al., 2003). Matching these predictions, flock size had a negative effect in peregrine hunt success. As expected, peregrine hunt success was also higher during low‐tide periods, when the overall abundance of sandpipers is lower in the shrimp farm (Basso et al., 2018).

Vigilance behavior recorded in western sandpipers was virtually absent in recently harvested (day 0) ponds, when foraging within flocks composed of several hundreds to thousands of individuals (Fonseca & Navedo, 2020). This indicates relative safe conditions by sharing vigilance when foraging in large flocks (e.g., Fernández & Lank, 2010), probably relying on “FEAR” behavior (after Blumstein, 2010). By contrast, sandpipers significantly increased their vigilance rate when foraging in small flocks at non‐optimal ponds, especially during neap tide periods when tidal cycles constrained availability of foraging areas (Fonseca et al., 2017). These results could reflect decisions made under a high starvation risk and limited opportunities, which tend to be individuals in poor condition that forage in small flocks (van den Hout et al., 2014). Since western sandpiper populations are male‐biased at this latitude (Nebel et al., 2002), these individuals would probably be males, particularly juveniles obliged to assume higher risk compared to adults (van den Hout et al., 2008). These few western sandpipers that remain at the shrimp farm rely on higher vigilance rates to reduce the comparatively higher per‐capita predation risk of foraging in smaller flocks (Cresswell & Quinn, 2010), in particular before the peak of the neap tide periods when overall abundance of sandpipers is also further reduced (Basso et al., 2018).

Noticeably, the overall abundance of western sandpipers at this wetland complex in a given season was strongly correlated with the observed attack rate by peregrine falcons. In addition, frequency of occurrence of peregrines at the shrimp farm was high, with peregrines being detected in nearly half of the surveys. Because peregrines observed in northern Mexico are most likely to be migratory individuals wintering during this period (White et al., 2020), our results reinforce the close relationship between migratory peregrines and shorebirds as prey (Ydenberg et al., 2004), especially western sandpipers, at wetlands where both predator and prey spend the nonbreeding season (Ydenberg et al., 2017). Since a variety of anthropogenic habitats provide alternative foraging areas used by shorebirds in different flyways, including semi‐intensive aquaculture operations (Jackson et al., 2019; Ma et al., 2010; Navedo et al., 2015; Watson et al., 2015), saltpans (Masero, 2003), and ricefields (Elphick, 2000), further studies should explore a potential link between availability of supratidal anthropogenic wetlands and the occurrence of migratory peregrine falcons during the non‐breeding season.

In conclusion, we found a strong link between predation risk by peregrine falcons on shorebirds at a shrimp farm that varies with foraging opportunities within the farm itself and with tidal conditions (amplitude and height) at the adjacent intertidal areas. During neap tide phases, starvation risk seems to overcome predation risk, and the main shorebird prey (western sandpipers) typically leave the wetland complex to go elsewhere (Basso et al., 2018), driving a drop in attack rate during consecutive days, irrespective of high‐ or low‐tide period. Our results therefore strongly support the consideration of moon phase (i.e., daily tidal amplitude) in future studies dealing with a trade‐off between starvation and predation risk for shorebirds at coastal wetlands. These monthly tidal phases, unlike the height of the tide (i.e., low and high tide; see Whitfield, 2003; Yasué et al., 2003; van de Hout et al., 2008), has not received much attention in previous predator–prey studies at these habitats. In addition, the relatively low hunt success we observed supports the notion that shorebirds can effectively avoid predation (van den Hout et al., 2014), supporting that indirect effects, including enhanced starvation risk (Houston & McNamara, 1999) driven by predictable restricted foraging opportunities, are important in life‐history decisions (Cresswell, 2008). Finally, our results indicate semi‐intensive shrimp farms embedded in wetlands can serve as an ideal experimental arena to study predator–prey interactions, given that foraging opportunities vary in a predictable manner according to tidal cycles and harvesting operations, both for sandpipers and peregrine falcons.

## CONFLICT OF INTEREST

The authors declare that they have no conflict of interest.

## AUTHOR CONTRIBUTIONS

**Enzo Basso:** Data curation (supporting); Formal analysis (supporting); Investigation (lead); Writing‐original draft (equal); Writing‐review & editing (equal). **Mark C. Drever:** Conceptualization (equal); Formal analysis (lead); Funding acquisition (equal); Project administration (supporting); Resources (supporting); Supervision (equal); Validation (equal); Visualization (equal); Writing‐original draft (equal); Writing‐review & editing (equal). **Juanita Fonseca:** Investigation (supporting); Project administration (supporting); Resources (supporting); Writing‐original draft (supporting); Writing‐review & editing (equal). **Juan G. Navedo:** Conceptualization (lead); Data curation (equal); Formal analysis (supporting); Funding acquisition (supporting); Investigation (equal); Methodology (lead); Project administration (lead); Resources (equal); Supervision (lead); Validation (lead); Visualization (equal); Writing‐original draft (equal); Writing‐review & editing (equal).

## STATEMENT OF HUMAN AND ANIMAL RIGHTS

All applicable institutional and/or national guidelines for the care and use of animals were followed.

## Data Availability

Dataset is fully accessible at https://datadryad.org/stash/dataset/doi:10.5061/dryad.f7m0cfxwt.
